# Serum Free Light Chains in Lymphoma: A Phenotype-First Framework for Biological Interpretation, Analytical Control, and Clinical Use

**DOI:** 10.3390/curroncol33100580

**Published:** 2026-09-28

**Authors:** Michele Bibas, Barbara Amoroso, Elena Matteini, Andrea Antinori, Valentina Mazzotta

**Affiliations:** 1 National Institute for Infectious Diseases, “Lazzaro Spallanzani,” IRCCS, Via Portuense 292, 00149 Rome, Italy; elena.matteini@inmi.it (E.M.); andrea.antinori@inmi.it (A.A.); valentina.mazzotta@inmi.it (V.M.); 2 Blue Wave Therapeutics GmbH, Kreuzstrasse 4, CH-4123 Allschwil, Switzerland; barbara.amoroso@bluewavetherapeutics.com

**Keywords:** serum free light chains, lymphoma, kappa/lambda ratio, monoclonal excess, polyclonal elevation, renal function, assay platform, chronic lymphocytic leukemia, diffuse large B-cell lymphoma, Waldenström macroglobulinemia

## Abstract

Serum free light chains (sFLC) reflect immunoglobulin production and renal clearance, making abnormal results biologically heterogeneous in lymphoma. We reviewed clinical, translational, and analytical evidence using a documented PubMed search. Three operational patterns should be distinguished: monoclonal-pattern elevation, polyclonal-pattern elevation, and ratio-only abnormality. The kappa-plus-lambda concentration is an overlapping measure of total circulating burden rather than a fourth exclusive phenotype. In diffuse large B-cell lymphoma, some cohorts reported International Prognostic Index-adjusted associations and improved model discrimination, whereas other studies did not confirm independent prognostic value. Evidence in classical Hodgkin lymphoma and chronic lymphocytic leukemia also depends on endpoint, adjustment, and treatment setting. Prediagnostic associations in human immunodeficiency virus infection, transplantation, and population cohorts do not establish screening utility. In Waldenström macroglobulinemia, involved-chain kinetics may supplement established assessment in selected patients, although clinical benefit from sFLC-directed management has not been demonstrated. Historical treatment regimens, overlapping cohorts, inconsistent renal adjustment, assay differences, and limited external validation restrict transferability. The proposed framework integrates phenotype, renal function, assay, sampling time, and disease context. It is an interpretive proposal, not a validated prognostic score or treatment algorithm.

## Simple Summary

Serum free light chain tests are established in plasma-cell disorders but are harder to interpret in lymphoma. An abnormal result can reflect secretion by a lymphoma clone, reactive antibody production, impaired renal clearance, or the assay itself. This review explains how kappa and lambda concentrations, the kappa/lambda ratio, kidney function, assay platform, and sampling time should be assessed together. Current studies support selected adjunctive interpretation at baseline and, in some patients with Waldenström macroglobulinemia, supplementary longitudinal assessment. The test does not independently diagnose lymphoma, define its subtype, replace biopsy or imaging, determine response, or guide treatment. Prediagnostic associations in human immunodeficiency virus infection, transplantation, and population cohorts remain research findings. Standardized reporting and prospective validation are required before broader use.

## 1. Introduction

Serum free light chains (sFLC) are unbound kappa and lambda immunoglobulin light chains generated during physiological antibody synthesis. Their concentrations reflect both production and clearance. Kappa chains usually circulate predominantly as monomers, lambda chains more often as dimers, and both are cleared mainly by glomerular filtration and proximal tubular catabolism [1,2,3,4,5]. This kinetic behavior explains the value of sFLC measurement in plasma-cell disorders, where involved-chain excess and kappa/lambda ratio distortion can reveal clonal secretion when intact immunoglobulin secretion is limited [2,3].

Lymphoma requires a different model. Most lymphomas are not primarily secretory diseases, and a numerically abnormal result can arise from several non-equivalent mechanisms: direct clonal secretion, plasmacytic differentiation, reactive B-cell or plasmablast activation, chronic antigenic stimulation, immunoparesis, renal retention, or analytical misclassification [2,5,6,7,8,9]. The clinically relevant question is therefore not whether sFLC is simply abnormal, but which phenotype is present and whether that phenotype is biologically plausible in the lymphoma context.

The biological source of an sFLC abnormality varies among lymphoma subtypes. In classical Hodgkin lymphoma (cHL) and many T-cell lymphomas, elevated sFLC is more likely to reflect reactive B-cell or plasma-cell activity within an inflammatory microenvironment [10,11,12,13]. Waldenström macroglobulinemia/lymphoplasmacytic lymphoma (WM/LPL) has a closer link between the malignant clone and secretory output [14,15,16,17,18,19]. Chronic lymphocytic leukemia/small lymphocytic lymphoma (CLL/SLL) combines clonal expansion with immune dysfunction [20,21,22,23,24], while diffuse large B-cell lymphoma (DLBCL) includes both secretory and inflammatory patterns and has produced conflicting prognostic results [25,26,27,28,29,30].

Lymphoma assessment increasingly incorporates histology, immunophenotype, molecular genetics, positron-emission tomography (PET), measurable residual disease in selected entities, and circulating tumor DNA (ctDNA) [31,32,33,34]. sFLC measures a different biological axis. It does not identify tumor mutations or viable tumor mass, but it may reflect secretory differentiation, systemic immune activation, or altered clearance.

An identical numerical result may therefore have different explanations across lymphoma entities, sampling times, and renal states. Interpretation should distinguish clonal secretion, polyclonal immune activation, and impaired clearance while accounting for assay platform and timing before any clinical inference.

This review examines the biological and clinical evidence for sFLC in lymphoma and proposes a practical sequence for interpretation. Diagnostic discrimination, prognostic association, prediction of differential treatment benefit, and evidence that testing improves management are treated as separate questions. The individual sFLC patterns are established concepts; their integration with renal, analytical, temporal, and entity-specific information has not been prospectively validated.

## 2. Methods and Scope

This article is a structured narrative review of the biological basis, analytical constraints, and clinical implications of sFLC measurement across adult lymphoid malignancies. The methods were informed by SANRA principles, including a stated aim, documented search logic, explicit selection criteria, and discussion of the limits of inference [35].

PubMed/MEDLINE was searched up to 17 June 2026 for records published from 1 January 1980 through that date, without a language restriction. The search combined title and abstract terms for free light chains with lymphoma, chronic lymphocytic leukemia or leukemia, Waldenström macroglobulinemia, and post-transplant lymphoproliferative disorders. The database search returned 226 records. The complete executable query and selection summary are provided in Supplementary Table S1.

Eligible primary evidence comprised human studies measuring circulating free light chains in lymphoma or CLL/SLL, clinically linked translational studies, and prediagnostic or transplant studies with a lymphoma-related endpoint. Analytical and renal-reference studies were retained when they changed interpretation. Case reports, conference abstracts, urine-only or cerebrospinal-fluid-only studies, and investigations without a relevant circulating-FLC result were not used to support clinical applications. Twenty-seven records from the PubMed search were included in the narrative synthesis; 199 were not selected after title and abstract screening. Additional analytical, biological, classification, and practice sources were identified by citation chaining and were not counted as a second exhaustive database search.

Selection and extraction were not independently duplicated. For each principal clinical report, we recorded the population, sample size, study design, recruitment period or treatment regimen, assay, abnormality definition, renal handling, endpoint, effect estimate, adjustment variables, and overlap with other publications. Unreported information was left unassigned rather than inferred. Negative findings were retained. Related reports were used for distinct analyses without counting overlapping patients as independent replication; the 2014 DLBCL analysis by Witzig et al., for example, reanalyzed part of the population reported by Maurer et al. [25,26].

Evidence was appraised qualitatively according to temporality, adjustment for established risk factors, renal confounding, assay comparability, missing data, cohort overlap, treatment era, and external validation. No numerical weighting scheme, formal risk-of-bias instrument, or certainty grade was applied. Supplementary Table S2 summarizes 27 reports: 26 original investigations and one narrative review by Kwapisz. The review provides contextual synthesis and is not an independent biomarker cohort. Study-level extraction and limitations are reported separately for each entry.

No pooled effect estimate was calculated because lymphoma classification, treatment era, assay platform, renal adjustment, phenotype definition, and endpoints were too heterogeneous for a meaningful meta-analysis. This review is a structured narrative synthesis and does not claim the completeness of a systematic review.

Scope boundaries were prespecified. Marginal zone lymphoma, Burkitt lymphoma, high-grade B-cell lymphoma, plasmablastic lymphoma, ALK-positive large B-cell lymphoma, primary effusion lymphoma, human herpesvirus 8 (HHV-8)-associated lymphoproliferative disorders, and primary CNS lymphoma are not developed as independent disease sections because lymphoma-specific serum sFLC data are sparse, heterogeneous, or largely case-based. These entities are discussed only when they clarify a biological boundary: chronic antigenic stimulation, plasmablastic or plasma-cell-like differentiation, HHV-8/HIV biology, or the distinction between serum biomarkers and compartment-specific cerebrospinal-fluid markers.

## 3. Biological Rationale: What an sFLC Abnormality Can Mean

### 3.1. Source, Modifier, and Timing

Interpretation begins with three questions: what could be producing the signal, what might modify the measurement, and when the sample was obtained. Potential sources include the lymphoma clone, reactive immune cells, a coincident monoclonal process, and renal retention. Important modifiers include estimated glomerular filtration rate (eGFR), assay platform, units, reference interval, dilution behavior, and antigen excess. Prediagnostic, pretreatment, on-treatment, and surveillance samples address different clinical questions.

This model explains why the same laboratory pattern can have different meanings. In a patient with WM/LPL, stable renal function, and a reproducible involved lambda excess, sFLC may approximate tumor-associated secretory activity. In a patient with cHL and preserved ratio, the same absolute elevation is more plausibly systemic immune activation. In a kidney-transplant recipient with falling eGFR, both kappa and lambda may rise primarily because of retention. Figure 1 summarizes this source-modifier-timepoint framework.

Physiological kinetics reinforce the need for context. Free light chains have short serum half-lives when renal function is preserved; kappa is generally cleared more rapidly than lambda, and both may change promptly when production or clearance changes [1,2,3,4,5]. Rapid movement is therefore not synonymous with rapid lymphoma response or progression. Infection, hydration, renal injury, immune reconstitution, and treatment toxicity can generate equally dynamic changes.

### 3.2. Operational Phenotypes

Three operational patterns should be distinguished. Cumulative kappa plus lambda is an additional quantitative measure that may overlap with any of the three patterns (Table 1).

Monoclonal excess describes elevation of at least one free light-chain type above its applicable reference interval, with the kappa/lambda ratio displaced in the direction of the elevated chain, after renal and assay context have been addressed. This pattern may suggest clonal secretion or plasmacytic differentiation, but it is not lymphoma-specific. Coexisting monoclonal gammopathy of undetermined significance (MGUS), occult plasma-cell disease, renal ratio distortion, antigen excess, and assay artifact remain competing explanations [2,5,6,7,8,9,36]. Confirmation should integrate serum protein electrophoresis (SPEP), immunofixation electrophoresis (IFE), immunoglobulin concentrations, histopathology, and reproducibility. 

Polyclonal elevation describes increased kappa and/or lambda with a preserved or renal-adjusted ratio. It is best interpreted as total humoral output, most often reflecting cytokine-driven B-cell or plasmablast activation, infection, chronic antigenic drive, autoimmune inflammation, immunocompromised-state biology, or renal retention. In cHL, T-cell lymphomas, HIV infection, and PTLD risk states, this is the default biological interpretation unless there is strong evidence for a separate monoclonal process [10,11,12,13,37,38,39,40,41,42,43,44].

Ratio-only abnormality is the least secure category. It denotes a kappa/lambda ratio outside the applicable reference interval with neither kappa nor lambda above its applicable upper reference limit. It may represent low-level clonal imbalance, but it is highly vulnerable to renal dysfunction, immunoparesis, suppression of the uninvolved light-chain compartment, age, platform thresholds, reference-interval selection, and stochastic variation near cut-offs [2,5,6,7,8,9]. In CLL/SLL this phenotype may be biologically plausible, but it should remain provisional until reproducible and concordant with the clinical picture.

The kappa-plus-lambda sum measures total circulating FLC burden rather than clonality. It may be elevated with monoclonal- or polyclonal-pattern abnormalities and remains strongly influenced by renal clearance. This measure has been evaluated most clearly in CLL/SLL [20,21]. Related total or bilateral elevations have been reported in HIV infection, transplantation, and prediagnostic NHL cohorts [37,38,43,44,45], but different populations, thresholds, and endpoints prevent direct equivalence. The sum should be reported separately and should not be used as a clonality marker or treatment trigger.

## 4. Analytical and Renal Constraints

### 4.1. The Ratio Is Not a Clonality Switch

sFLC immunoassays detect epitopes exposed on free kappa and lambda chains and largely hidden in intact immunoglobulins [2,3,6]. This analytical sensitivity is useful in secretory plasma-cell diseases, but in lymphoma it increases vulnerability to low-level, borderline, or context-dependent abnormalities. The kappa/lambda ratio is a derived variable influenced by production and clearance of both chains. A slightly abnormal ratio near a decision threshold is not equivalent to marked involved-chain excess in a secretory clone, and a normal ratio does not exclude biologically relevant polyclonal activation or high cumulative burden.

### 4.2. Renal Function Is the Dominant Physiological Confounder

Renal function is the dominant non-tumor determinant of sFLC concentration. As glomerular filtration declines, both kappa and lambda increase, and the kappa/lambda ratio may shift upward without monoclonal secretion [4,5]. The iStopMM chronic-kidney-disease analysis showed that standard reference intervals generate frequent apparent abnormalities among individuals without evidence of monoclonality. eGFR-stratified FLC ratio intervals were proposed: 0.46–2.62 for eGFR 45–59 mL/min/1.73 m^2^, 0.48–3.38 for eGFR 30–44 mL/min/1.73 m^2^, and 0.54–3.30 for eGFR below 30 mL/min/1.73 m^2^ [5].

These intervals should not be applied mechanically across lymphoma populations or assay platforms. Creatinine, eGFR, and renal trajectory should accompany every clinically interpreted sFLC result. Stable chronic kidney disease (CKD), acute kidney injury during infection, tumor lysis, dehydration, nephrotoxic therapy, and graft dysfunction are different settings; borderline ratios are particularly unreliable when renal function is reduced or changing.

Population studies further show how reference-interval selection changes classification. In iStopMM, age- and kidney-appropriate intervals reduced the apparent prevalence of light-chain monoclonal gammopathy of undetermined significance (LC-MGUS) by 82%; none of 1006 reclassified individuals developed a lymphoproliferative disorder during a median of 4.6 years [46]. Danish validation reduced LC-MGUS diagnoses by about 40%; none of the reclassified participants developed multiple myeloma or immunoglobulin light-chain (AL) amyloidosis, although two developed non-Hodgkin lymphoma [47]. These are not lymphoma cohorts, but they support cautious interpretation of borderline ratios using age-, kidney-, and method-appropriate intervals.

### 4.3. Platforms Are Not Interchangeable

Freelite, Siemens N Latex, Sebia, enzyme-linked immunosorbent assays, and emerging methods differ in antibody specificity, calibration, dynamic range, dilution behavior, susceptibility to antigen excess, and reference intervals [6,7,8,9,48]. A result near a decision threshold can therefore change category when another method is used.

Serial measurements should use the same platform whenever possible. A method change establishes a new analytical baseline; parallel testing may be needed when continuity matters. Historical prevalence estimates and cut-offs should not be transferred directly across assays.

Mass-spectrometric methods can help distinguish monoclonal from polyclonal immunoglobulin signals and clarify discordant conventional results. However, light chains released from immunoenriched intact immunoglobulins are not identical to directly measured circulating free light chains. Lymphoma-specific prognostic and monitoring performance has not been established, so these methods remain a research approach in this setting [6].

### 4.4. Units, Reference Intervals, Dilution, and Antigen Excess

Reference intervals are method-specific and population-dependent. The commonly cited Freelite kappa/lambda interval of approximately 0.26–1.65 is not a universal biological constant [3]. It should not be transferred uncritically to other platforms, CKD populations, or lymphoma cohorts. Unit reporting is equally important: mg/dL and mg/L differ by a factor of ten, and silent conversion errors can alter apparent thresholds.

Antigen excess is less frequent but clinically important. Very high free light-chain concentrations can produce falsely low or implausibly normal values unless adequate dilution protocols are triggered [6,36]. In lymphoma, this matters most in WM/LPL, plasmacytic or plasmablastic differentiation, marked secretory behavior, and coexisting monoclonal gammopathy. Discordance between sFLC, SPEP/IFE, immunoglobulin levels, IgM, marrow disease, imaging, or clinical behavior should prompt laboratory verification rather than a biological conclusion.

### 4.5. Minimum Reporting Dataset

An interpretable report should include kappa, lambda, kappa/lambda ratio, units, assay platform, reference intervals, creatinine, eGFR, renal-adjusted interpretation when relevant, phenotype, timing of sampling, treatment context, and assessment for coexisting monoclonal gammopathy when a monoclonal pattern is present. Without these elements, a reader cannot distinguish tumor secretion from host activation, renal retention, ratio distortion, or analytical artifact. The minimum reporting dataset and the interpretive rationale for each required element are summarized in Table 2.

Clinical laboratories can reduce misuse through interpretive safeguards. Reflex comments may state that absolute elevations require renal and inflammatory context, that ratio-only abnormalities are less secure when eGFR is reduced, and that serial comparisons require the same platform. Such comments are particularly useful outside plasma-cell practice, where the kappa/lambda ratio may otherwise be misread as a binary clonality switch.

## 5. Evidence Across Lymphoma Entities

Evidence and biological interpretation differ among lymphoma entities. WM/LPL provides the clearest secretory rationale; CLL/SLL includes several phenotype-resolved associations; cHL and T-cell lymphomas more often show reactive immune activation; and immunocompromised cohorts inform prediagnostic risk. Mixed-entity and small relapsed cohorts remain hypothesis-generating [49,50]. Table 3 summarizes the principal studies and their main limits.

### 5.1. Diffuse Large B-Cell Lymphoma

Maurer et al. studied 295 patients with DLBCL in two cohorts. Elevated sFLC was associated with event-free survival (EFS) and overall survival (OS) after adjustment for the International Prognostic Index (IPI): combined hazard ratios were 2.37 (95% confidence interval [CI], 1.58–3.55) and 2.94 (95% CI, 1.85–4.77), respectively [25]. Adding sFLC increased the reported 36-month C-index from 0.606 to 0.667 for EFS and from 0.658 to 0.721 for OS. These results show incremental discrimination within that dataset; they do not establish external calibration, treatment prediction, or clinical benefit from sFLC-guided management.

Witzig et al. subsequently analysed 276 patients from part of the same source population, with outcome analyses in 256 patients receiving immunochemotherapy [26]. Both monoclonal- and polyclonal-pattern elevations were associated with inferior EFS, and monoclonal-pattern cases were enriched for non-germinal-centre phenotypes by immunohistochemical algorithms. This report adds phenotype and biological detail but is not an independent replication of the earlier cohort.

Independent reports are not uniform. Kim et al. studied 175 patients treated with R-CHOP and reported an IPI-adjusted EFS association for elevated FLC (HR 2.84; 95% CI, 1.29–6.25) [27]. Maiolo et al. selected 61 patients with a serum monoclonal protein and 45 IPI-matched controls; the kappa-associated EFS signal was observed among controls without monoclonal protein, not as equivalent prognostic information within the selected monoclonal-protein group [28]. Achenbach et al. found no IPI-adjusted EFS or OS association in 259 RICOVER-60 patients with eGFR at least 60 mL/min/1.73 m^2^, and the negative result was reproduced in an independent 155-patient rituximab-treated cohort [30].

Jia et al. included 259 patients diagnosed in 2017–2022; 191 receiving R-CHOP entered survival analyzes and 68 were lost to follow-up [29]. Abnormal FLC was associated with lower unadjusted three-year OS and progression-free survival, but independent prognostic significance was not established. These cohorts largely reflect CHOP-based therapy and do not validate an sFLC-based decision rule for current frontline combinations, cellular therapy, or bispecific antibodies. sFLC therefore remains an exploratory adjunct in DLBCL.

### 5.2. Classical Hodgkin Lymphoma

In cHL, polyclonal elevation is the predominant pattern and is consistent with an inflammatory microenvironment [10,11]. Thompson et al. analyzed 100 patients, including 93 with cHL. Elevated sFLC retained an association with EFS after adjustment for the International Prognostic Score (HR 3.02; 95% CI, 1.02–8.89), whereas adjusted OS could not be estimated because only 12 deaths occurred [11]. The study therefore supports an adjusted EFS association, not an independently adjusted OS prediction.

The incremental value of sFLC alongside PET-based assessment and modern regimens is unknown [34,51,52]. Baseline polyclonal sFLC may describe inflammatory biology, but current evidence does not support its use to alter interim PET interpretation, treatment intensity, or response classification.

### 5.3. Follicular Lymphoma and Marginal Zone Lymphoma

Follicular lymphoma (FL) is a lower-signal setting. Available evidence suggests relatively infrequent abnormalities, often polyclonal, and much of the early evidence comes from mixed B-cell lymphoma cohorts [50,53]. The Follicular Lymphoma International Prognostic Index, tumor burden, histological transformation risk, PET features, and molecular pathology remain more clinically relevant [54]. A monoclonal sFLC pattern in FL should not be assumed to represent ordinary FL biology; it should prompt evaluation for plasmacytic differentiation, composite disease, transformation, MGUS, or a coexisting plasma-cell clone [55].

Marginal zone lymphoma is biologically interesting because of chronic antigenic stimulation, autoimmune disease, infection-associated lymphomagenesis, and plasmacytic differentiation. However, robust entity-specific serum sFLC datasets remain insufficient for routine recommendations. In both FL and marginal zone lymphoma, sFLC should be a contextual clue, not a risk-stratification system.

### 5.4. Chronic Lymphocytic Leukemia/Small Lymphocytic Lymphoma

Maurer et al. prospectively evaluated 339 newly diagnosed patients with CLL/SLL and distinguished monoclonal-pattern, polyclonal-pattern, and ratio-only abnormalities [20]. In an adjusted model, monoclonal-pattern elevation was associated with time to first treatment (HR 2.97; 95% CI, 1.75–5.06), while elevated FLC combining monoclonal and polyclonal patterns was associated with OS (HR 2.25; 95% CI, 1.04–4.86). Morabito et al. studied 449 treatment-naive patients; cumulative kappa plus lambda retained an association with treatment-free survival (HR 2.3; 95% CI, 1.3–4.1) in a model that also included the ratio and established risk factors [21]. The reported concentration units are internally inconsistent, which prevents direct adoption of the numerical threshold.

These cohorts do not establish treatment prediction or clinical utility. Modern Bruton tyrosine kinase and BCL2-directed therapies alter tumor burden, lymphocyte distribution, immunoglobulin recovery, and infection risk [56]. sFLC should be evaluated again in those settings and should not replace the International Workshop on CLL treatment criteria, genetic risk assessment, or measurable residual disease methods [57].

Prediagnostic and familial studies indicate that immunoglobulin abnormalities may precede overt CLL/SLL or occur in monoclonal B-cell lymphocytosis and high-risk families [22,23,24]. These findings concern early biological states and do not make sFLC a treatment trigger or population-screening test.

### 5.5. Mantle Cell Lymphoma

Mantle cell lymphoma (MCL) shows heterogeneous sFLC biology. An abnormal ratio was associated with poor overall survival in a relapsed/refractory MCL series, and mixed-entity non-Hodgkin lymphoma data suggest that abnormalities are not rare [49,50]. These observations are biologically plausible in leukemic-phase, marrow-involved, or secretory subsets, but evidence remains small and treatment-era-specific.

Current MCL care increasingly incorporates morphology, Ki-67, TP53 status, the Mantle Cell Lymphoma International Prognostic Index (MIPI), leukemic non-nodal phenotype, BTK inhibitors, CAR-T therapy, BCL2-directed approaches, non-covalent BTK inhibition, and T-cell-engaging therapies [58]. sFLC kinetics are not validated in this setting. A markedly abnormal, reproducible ratio may enrich for higher-risk biology in selected patients, but it should not guide treatment outside research.

### 5.6. Waldenström Macroglobulinemia/Lymphoplasmacytic Lymphoma

WM/LPL offers the clearest biological rationale for a secretory adjunct. Itzykson et al. studied 42 untreated patients and found involved FLC associated with time to treatment; 28 patients were already symptomatic at baseline [14]. Leleu et al. linked sFLC to burden-related features in 98 patients [15]. In a prospective treatment study of 48 patients, an involved-FLC response occurred in 79%, compared with 60% by M-spike criteria [16]. Among 27 patients responding by both measures, the median response time was 2.1 months for FLC and 3.7 months for the M-spike. The agreement was only fair, and the landmark FLC response was not significantly associated with time to progression.

Earlier biochemical change is not evidence that earlier intervention improves outcome. Selective serial use is most reasonable when the baseline involved-chain abnormality is reproducible, renal function is stable, and the same assay is used. IgM, M-spike, symptoms, marrow or tissue disease, and the International Workshop on Waldenström Macroglobulinemia criteria remain the basis for clinical decisions [17,18,19].

### 5.7. T-Cell Lymphomas

In T-cell lymphomas, elevated sFLC is more likely to reflect reactive B-cell or plasma-cell activation, infection, Epstein–Barr virus-associated B-cell expansion, or cytokine-driven immune dysregulation than direct secretion by malignant T cells [12,13]. This interpretation is particularly relevant to nodal T-follicular-helper lymphomas, including angioimmunoblastic T-cell lymphoma.

Current evidence does not support routine sFLC testing as an independent prognostic biomarker in T-cell lymphomas. A convincing monoclonal sFLC pattern should prompt evaluation for a coexisting B-cell or plasma-cell process rather than being attributed automatically to the T-cell lymphoma.

### 5.8. HIV-Associated Lymphomas

In HIV-associated lymphoma, timing is decisive. Landgren et al. showed that elevated sFLC can precede AIDS-related NHL, and Bibas et al. linked sFLC to HIV-associated lymphoma risk and antiretroviral-therapy context [37,38]. These data support a chronic immune-activation, risk-state model. In contrast, diagnostic-phase studies indicate that sFLC does not reliably classify histology or prognosis once lymphoma is established [39].

HIV viral load, CD4 count, ART exposure, EBV and HHV-8 biology, microbial translocation, opportunistic infection, immune reconstitution, and renal function can all affect kappa and lambda concentrations [37,38,39,40,41,42]. Prediagnostic and diagnostic samples should not be merged conceptually. Before lymphoma, elevated sFLC may reflect immune activation that contributes to lymphomagenesis; at diagnosis, the same abnormality may reflect infection, viremia, renal dysfunction, lymphoma burden, or treatment context. A small exploratory study suggested that Epstein–Barr virus load and sFLC kinetics might have follow-up value [42], but this has not been prospectively validated and remains vulnerable to infectious, renal, and treatment-related confounding. sFLC is therefore best considered a research-level immune-activation and risk-state marker, not a diagnostic classifier or routine surveillance test.

### 5.9. Post-Transplant Lymphoproliferative Disorder

PTLD studies address different time points. Engels et al. measured samples obtained before diagnosis and found an association between polyclonal-pattern elevation and subsequent PTLD [43]. Borrows et al. measured sFLC at diagnosis in transplant recipients and showed that adjustment to cystatin C strengthened the association [44]. The latter is diagnostic-phase evidence, not prediagnostic prediction. Renal dysfunction, graft type, infection, immunosuppression, and Epstein–Barr virus status limit direct comparison [59].

EBV DNAemia and sFLC are complementary rather than interchangeable. EBV DNAemia reflects viral or EBV-infected B-cell burden, whereas sFLC may reflect host B-cell activation and impaired immune control. Neither replaces tissue diagnosis when PTLD is suspected. Pediatric PTLD biomarker consensus documents are useful for monitoring principles but should not be treated as adult lymphoma practice evidence [60].

### 5.10. Prediagnostic Risk-State Biology Beyond Immunocompromised Hosts

Hu et al. examined prediagnostic samples from 292 incident NHL cases and matched controls in the Prostate, Lung, Colorectal and Ovarian Cancer Screening Trial [45]. Higher kappa and lambda concentrations were associated with subsequent NHL. Among CLL/SLL cases, polyclonal elevations were more common in samples collected farther from diagnosis and monoclonal patterns in samples collected closer to diagnosis.

This cross-group timing pattern is compatible with changing immune and clonal states, but it does not demonstrate within-person progression from a polyclonal to a monoclonal abnormality. Population screening would require validated thresholds, absolute-risk estimates, acceptable positive predictive value, and evidence that subsequent action improves outcomes.

### 5.11. Cross-Entity Synthesis

Across entities, the meaning of sFLC follows the biological question. Secretory abnormalities are most plausible in WM/LPL; CLL/SLL includes clonal and immune-dysregulation patterns; cHL and T-cell lymphomas more often reflect reactive immune activation; and DLBCL contains mixed mechanisms with inconsistent prognostic evidence. HIV, PTLD, and population cohorts inform prediagnostic risk research, not clinical screening. Table 4 summarizes the current interpretive role and the principal limitation in each setting.

Evidence remains sparse for marginal zone lymphoma, Burkitt lymphoma, high-grade B-cell lymphoma, plasmablastic lymphoma, ALK-positive large B-cell lymphoma, primary effusion lymphoma, HHV-8-associated lymphoproliferative disorders, and primary central nervous system lymphoma. Their biology may justify study, but current data do not support routine serum sFLC recommendations. Cerebrospinal-fluid free light chains in central nervous system disease address a compartment-specific question and should not be equated with serum results.

## 6. Clinical Applications and Boundaries

### 6.1. Selective Use Only

The current evidence supports selective interpretation when there is a defined clinical question. Potential uses include baseline biological context in DLBCL, cHL, and CLL/SLL; supplementary serial assessment in selected secretory WM/LPL; and risk-state research in immunocompromised or prediagnostic cohorts [11,14,15,16,20,21,25,26,27,28,29,30,37,38,39,43,44,45]. None has been validated as a universal testing strategy.

An independent prognostic association means that a marker retained an association within a particular model. It does not establish the prediction of treatment benefit or clinical utility. The DLBCL discrimination analysis provides a positive example, while the null DLBCL cohort and the limited diagnostic-phase HIV findings show why results cannot be generalized [25,30,39]. No study reviewed here demonstrated improved outcomes from changing lymphoma management according to sFLC.

### 6.2. Pattern-Specific Clinical Interpretation

For a monoclonal-pattern elevation, review the direction and magnitude of the ratio, the absolute involved-chain concentration, renal function, and assay-specific limits. Correlate the result with serum protein electrophoresis, immunofixation, immunoglobulins, and tissue findings before attributing it to lymphoma [2,3,4,5,6,7,8,9,27,28]. A coexisting monoclonal gammopathy or plasma-cell clone remains possible.

For a polyclonal-pattern elevation, consider renal clearance together with infection, autoimmune disease, HIV, transplantation, and tumor-associated inflammation [4,5,10,11,12,13,37,38,39,40,43,44]. A preserved ratio supports balanced production but does not identify the cause.

A ratio-only abnormality deserves particular caution near decision limits and when renal function is reduced [3,4,5,6,7,8,9]. Repeat measurement under stable conditions may help, but reproducibility alone does not establish clonality. Cumulative kappa plus lambda should be reported separately with units and renal context [20,21,38,44].

Reports and multidisciplinary discussions should describe what the pattern supports and what remains uncertain. Appropriate wording may state that a result is concordant with a secretory phenotype, compatible with polyclonal immune activation, or difficult to interpret because renal function is changing. A diagnostic or treatment conclusion requires corroborating evidence.

Figure 2 presents the interpretive sequence: define the clinical question, verify assay and renal context, assign one of the three operational patterns, record kappa plus lambda separately, and then consider disease entity and sampling time. The sequence is an expert interpretive proposal and has not been prospectively tested as a package.

### 6.3. Longitudinal Monitoring

Longitudinal monitoring should be restricted mainly to WM/LPL and rare lymphoma cases with a reproducible, disease-linked monoclonal abnormality. Even in WM/LPL, sFLC is an adjunct and does not replace IgM-based response criteria, M-spike, marrow assessment, tissue disease evaluation, symptoms, or hyperviscosity risk [17,18,19]. In DLBCL and cHL, PET/CT-based response assessment and disease-specific criteria remain primary [34].

A serial change is uninterpretable if the assay platform changed, eGFR changed substantially, acute infection intervened, dilution behavior was not verified, or the baseline phenotype was not defined. The short half-life of free light chains does not make every lymphoma suitable for sFLC monitoring.

### 6.4. Prediagnostic and High-Risk Contexts

Prediagnostic sFLC elevation in HIV infection, PTLD, and population-based NHL cohorts is scientifically important because it supports immune risk-state biology [37,38,43,44,45]. It remains research-facing. In clinical practice, symptoms, lymphadenopathy, organ involvement, EBV DNAemia, uncontrolled HIV replication, or unexplained inflammatory or renal changes—not sFLC alone—should drive reassessment; lymphoma still requires tissue diagnosis.

The workflow is intended to prevent both indiscriminate dismissal of sFLC abnormalities and unsupported clinical action. It preserves potentially useful biological information while requiring corroboration before diagnosis, response assessment, surveillance, or treatment decisions. The practical stopping rules are summarized in Box 1.

Box 1 **Clinical rules for sFLC use in lymphoma.**

- Start with phenotype, not abnormality.
- Measure kappa, lambda, kappa/lambda ratio, creatinine, and eGFR together.
- Use renal-contextualized interpretation when eGFR is reduced or unstable.
- Do not infer clonality from absolute kappa or lambda elevation alone.
- Do not compare serial values across assay platforms or laboratories without treating the change as a new baseline.
- Reserve monitoring for reproducible disease-linked abnormalities, mainly WM/LPL.
- Do not replace biopsy, PET/CT, MRD, ctDNA, IWWM, iwCLL, Lugano, or other disease-specific criteria.
- Do not initiate, escalate, de-escalate, or stop therapy on sFLC alone.

## 7. Limitations of the Evidence

Most lymphoma studies are observational, many are retrospective, and several arise from overlapping populations. Historical treatment regimens limit applicability to current care. Associations with outcomes do not show that sFLC-guided management improves survival, response, quality of life, diagnostic efficiency, or surveillance.

Studies differ in lymphoma classification, disease stage, sampling time, assay platform, renal handling, endpoint, phenotype definition, and adjustment set. Platform differences can alter concentrations and classification [6,7,8,9,48], while chronic or acute kidney dysfunction can produce polyclonal elevations and borderline ratio shifts [4,5,46,47]. Restriction to normal creatinine, use of a renal ratio interval, and statistical adjustment for renal function address different problems.

Terminology is also inconsistent. Some studies define an abnormal ratio as monoclonal, whereas others require involved-chain elevation. Endpoints such as time to first treatment, EFS, OS, biochemical response, and future lymphoma risk are not interchangeable. Prediagnostic associations remain unsuitable for screening because absolute risk, specificity, positive predictive value, and an effective intervention pathway have not been established.

This review has the limitations of a single-database narrative search. Selection and extraction were not independently duplicated, no formal risk-of-bias instrument was applied, and some reports did not provide complete assay, renal, or modelling details. Heterogeneity precluded meta-analysis. The proposed framework has not undergone prospective or interobserver validation.

## 8. Research Agenda

Future studies should prespecify the clinical question, lymphoma entity, treatment setting, and endpoint. They should record kappa, lambda, ratio, kappa plus lambda, assay, units, reference intervals, creatinine, eGFR, renal trajectory, infection status, and coexisting monoclonal proteins. Pattern assignment should be defined before analysis, and continuous modelling should accompany any proposed cut-off.

Entity-specific cohorts should compare standard models with and without sFLC. DLBCL studies should incorporate IPI, cell of origin, MYC/BCL2/BCL6 genetics, PET metrics, inflammatory markers, and ctDNA; cHL studies should use contemporary regimens and PET-based assessment; CLL/SLL studies should integrate genomic risk and measurable residual disease; and WM/LPL studies should examine paired IgM, involved-FLC, renal, and clinical trajectories. Calibration, discrimination, external validation, and decision-curve analyses should be reported.

Mechanistic studies can link sFLC to immune-cell states, cytokine networks, B-cell receptor repertoire, Epstein–Barr virus and HHV-8 biology, renal biomarkers, and ctDNA. Such analyses could determine whether sFLC adds information beyond inflammatory burden, secretory differentiation, renal function, or tumor mass. The framework itself should also be tested for interobserver agreement before its effect on management is studied.

Future studies should prespecify the clinical decision that sFLC is intended to improve and include negative as well as positive results. The minimum reporting and design requirements are summarized in Box 2.

Box 2 **Minimum design requirements for future lymphoma sFLC studies.**

- Prespecify the clinical question and endpoint before measuring sFLC.
- Report kappa, lambda, ratio, units, platform, reference interval, creatinine, eGFR, and renal trajectory.
- Code phenotype at patient level instead of using one pooled abnormal/normal variable.
- Document SPEP, IFE, immunoglobulins, and MGUS/plasma-cell evaluation when monoclonal patterns occur.
- Use the same platform for serial analysis and handle platform changes as new baselines.
- Validate incremental value against disease-specific clinical, imaging, molecular, and response tools.
- Report null and non-independent findings to reduce publication bias.

## 9. Conclusions

sFLC results in lymphoma are interpretable only when the operational pattern, renal function, assay platform, sampling time, and disease entity are considered together. Monoclonal-pattern elevation, polyclonal-pattern elevation, and ratio-only abnormality describe different findings; kappa plus lambda is an overlapping measure of total circulating burden.

Current evidence supports selected adjunctive interpretation at baseline and supplementary longitudinal assessment in some patients with WM/LPL. Prognostic independence is inconsistent, and no study has shown improved outcomes from an sFLC-directed lymphoma management strategy. sFLC should not independently establish diagnosis, subtype, response, relapse, screening eligibility, or treatment.

Prospective validation is now required in phenotype-defined, renal-adjusted, assay-consistent contemporary cohorts. Studies must show added prediction and a clinically useful decision benefit before sFLC can be adopted more broadly. Clinical utility also requires reproducible phenotype assignment and consistent recognition of confounders.

## Figures and Tables

**Figure 1:**
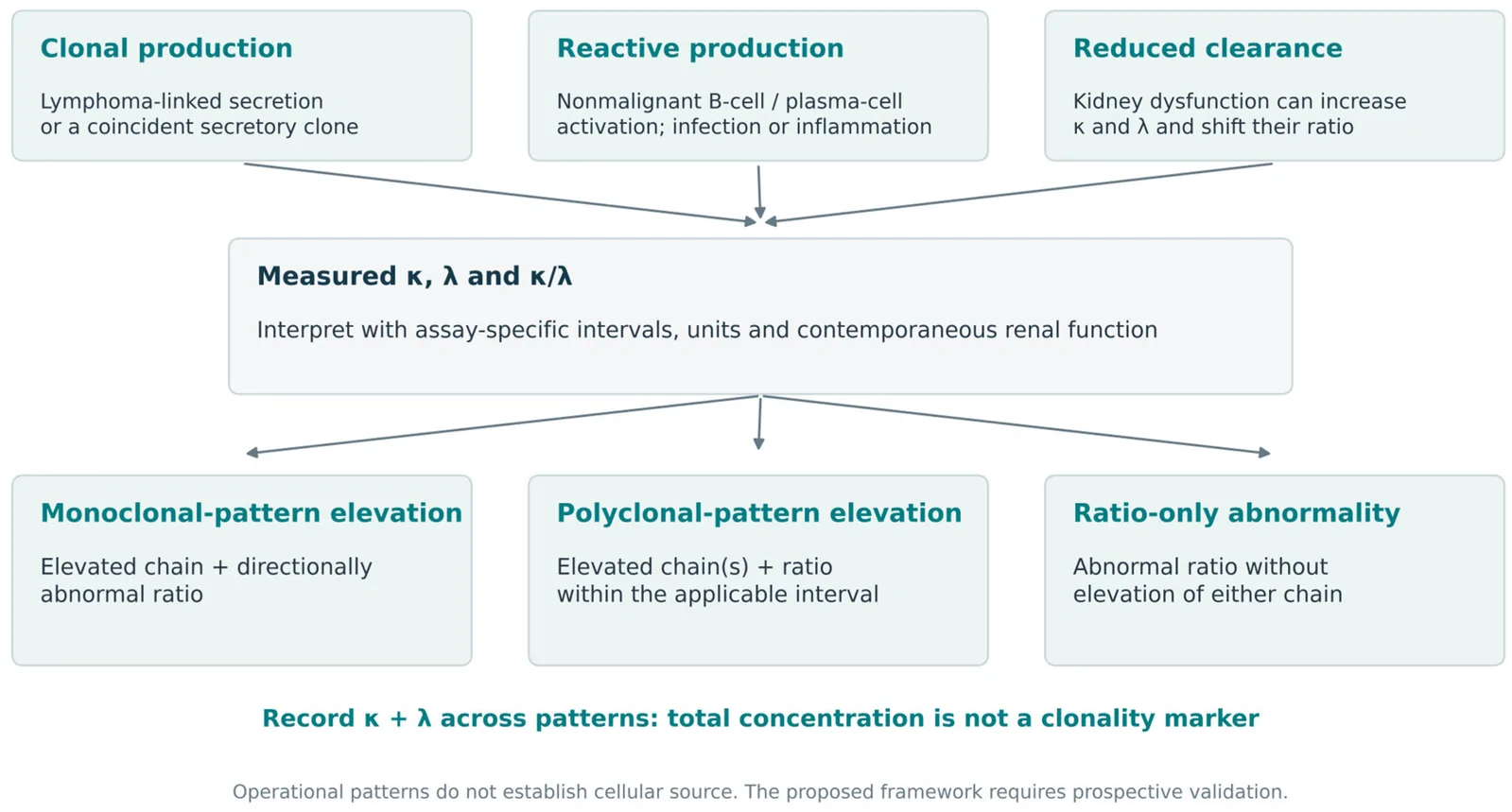
Biological sources and modifiers of serum free light-chain measurements in lymphoma. Production and renal clearance jointly determine kappa and lambda concentrations. Assay characteristics and sampling time should be reviewed before assigning an operational pattern or drawing a clinical inference.

**Figure 2:**
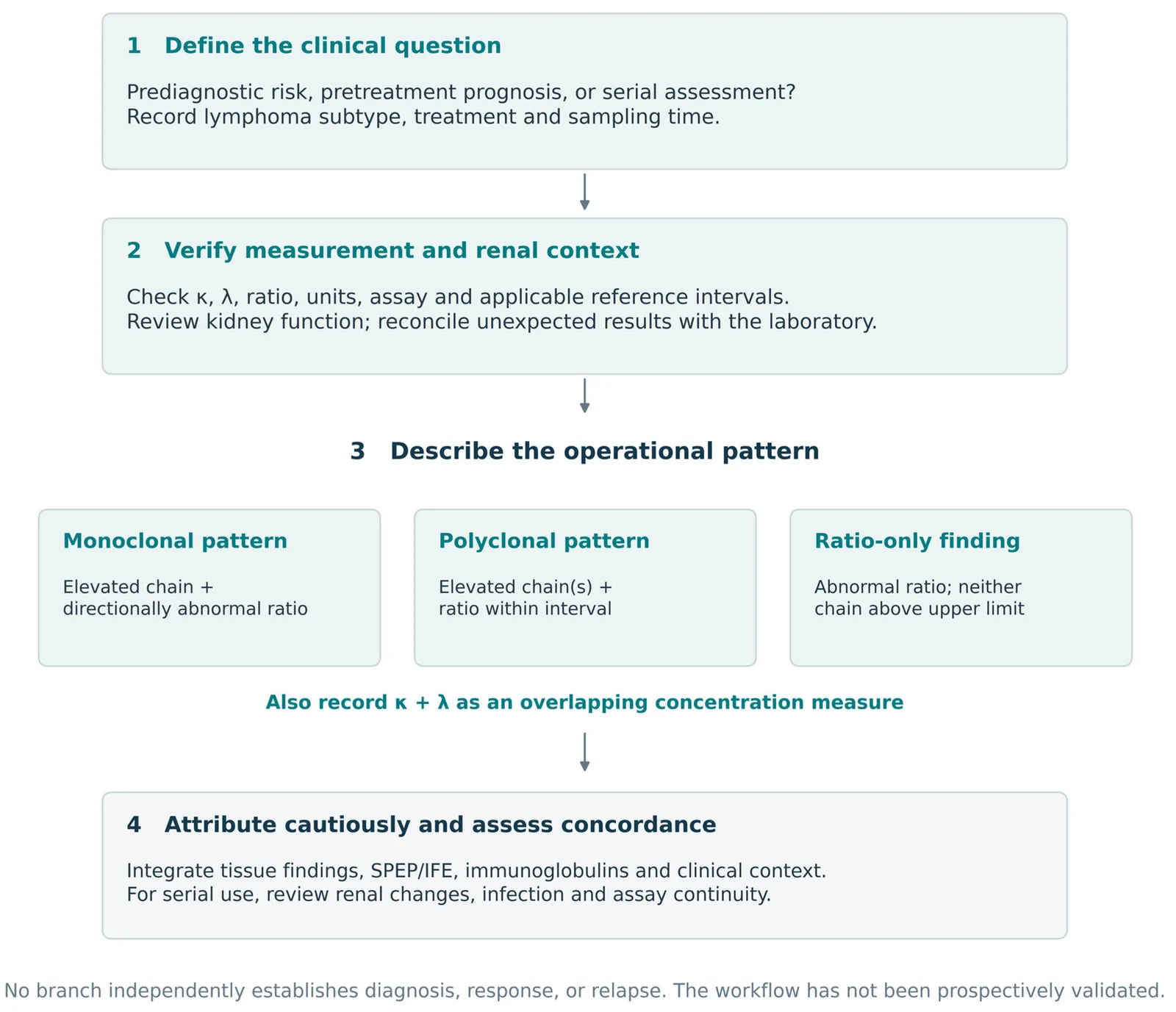
Proposed workflow for interpreting serum free light chains in lymphoma. Laboratory and renal checks precede assignment of an operational pattern. Clinical interpretation then depends on disease entity, sampling time, and the strength of the supporting evidence. Kappa plus lambda is recorded as an overlapping quantitative measure rather than an exclusive phenotype.

**Table 1:** Operational phenotype definitions for sFLC interpretation in lymphoma.

Phenotype	Operational Definition	Most Likely Biology	Immediate Safeguard
Monoclonal excess	At least one chain above its applicable upper reference limit, with the κ/λ ratio displaced toward that chain after renal and assay context are considered.	Clonal secretion, plasmacytic differentiation, WM/LPL, selected B-cell lymphomas; possible MGUS or plasma-cell clone.	Confirm eGFR, assay, SPEP/IFE, immunoglobulins, histology, and reproducibility before attribution to lymphoma.
Polyclonal elevation	κ and/or λ above reference interval with preserved or renal-adjusted ratio.	Global humoral activation, cytokine-driven B-cell/plasmablast output, infection, HIV, PTLD biology, inflammation, renal retention.	Do not infer clonality from absolute elevation alone; review inflammatory, infectious, renal, and immune context.
Ratio-only abnormality	κ/λ ratio outside the applicable reference interval, with neither κ nor λ above its applicable upper reference limit.	Low-level clonal imbalance, immunoparesis, suppressed uninvolved compartment, renal shift, platform threshold effect.	Treat as provisional; repeat on same platform when renal function is stable and correlate with secretory studies.
Cumulative kappa + lambda (overlapping measure)	Sum of kappa and lambda concentrations, recorded across the three operational patterns.	Total circulating FLC burden from production and clearance; evaluated in CLL/SLL and prediagnostic studies.	Report separately with units and renal context; do not use as a clonality marker or treatment trigger.

Abbreviations: eGFR, estimated glomerular filtration rate; IFE, immunofixation electrophoresis; MGUS, monoclonal gammopathy of undetermined significance; SPEP, serum protein electrophoresis; WM/LPL, Waldenström macroglobulinemia/lymphoplasmacytic lymphoma.

**Table 2:** Minimum reporting dataset for interpretable sFLC use.

Domain	Required Reporting Item	Why It Matters
Assay	Platform, manufacturer, method, units, and reference intervals.	Prevents method transfer, unit conversion error, and false continuity across laboratories.
Renal context	Creatinine and eGFR at sampling; renal trajectory; renal-adjusted interpretation when relevant.	Controls the dominant non-tumor determinant of sFLC concentration and ratio shift.
Phenotype	Monoclonal excess, polyclonal elevation, ratio-only abnormality, and/or cumulative κ + λ burden.	Prevents biologically different mechanisms from being pooled as one abnormal result.
Clinical timing	Prediagnostic, baseline, on-treatment, post-treatment, surveillance, or relapse sample.	The same result has different meanings at different time points.
Disease context	Entity, classification, stage, treatment era, established risk tools, PET/MRD/ctDNA context when relevant.	Anchors interpretation to lymphoma-specific biology and standards of care.
Secretory context	SPEP, IFE, immunoglobulins, IgM/M-spike in WM/LPL, MGUS/plasma-cell assessment when indicated.	Separates lymphoma-associated secretion from a coexisting monoclonal or plasma-cell process.
Technical safeguards	Dilution behavior, antigen-excess handling, sample timing, and laboratory/platform changes.	Protects against false reassurance and non-comparable serial values.

**Table 3:** Principal clinical evidence map.

Study	Setting	Principal Signal	Boundary for Use
Maurer et al. 2011 [25]	DLBCL cohorts	IPI-adjusted EFS HR 2.37 and OS HR 2.94; reported C-index improvement within the study dataset.	Incremental discrimination in one dataset; no external decision-benefit validation.
Witzig et al. 2014 [26]	Untreated DLBCL	Monoclonal and polyclonal FLC, with interferon-inducible protein 10 (IP-10), linked to inferior prognosis.	Partly overlaps the Maurer population and is not an independent replication.
Kim et al. 2014; Maiolo et al. 2020 [27,28]	DLBCL	Kim reported an adjusted EFS association; Maiolo found a kappa-associated EFS signal only among controls without monoclonal protein.	Different selection criteria and phenotype definitions preclude a single conclusion.
Jia et al. 2025 [29]	Newly diagnosed DLBCL	Unadjusted outcome differences were reported; independent prognostic value was not established.	Independent multivariable prognostic value was not established.
Achenbach et al. 2014 [30]	Elderly aggressive CD20-positive B-cell lymphoma	No IPI-adjusted EFS or OS association in 259 patients with preserved eGFR; negative result reproduced in 155 patients.	Strong non-confirmatory evidence; age- and renal-selected population.
Thompson et al. 2011 [11]	cHL	IPS-adjusted EFS association; adjusted OS was not estimable because only 12 deaths occurred.	Single historical cohort; not validated for PET-adapted decisions.
Maurer et al. 2011; Morabito et al. 2011 [20,21]	CLL/SLL	Monoclonal/polyclonal patterns and cumulative FLC burden associated with TTFT/outcome.	Adjunctive risk context; does not replace iwCLL criteria or MRD.
Furtado et al. 2013 [49]	Relapsed/refractory MCL	Abnormal FLC ratio associated with poor overall survival.	Small, treatment-era-specific; not validated in contemporary MCL therapy.
Itzykson, Leleu and colleagues [14,15,16]	WM/LPL	Associations with burden and time to treatment; earlier FLC response among 27 dual responders.	Fair response agreement and no significant landmark association with time to progression.
Witzig et al. 2014 [50]	Mixed B- and T-cell NHL	Monoclonal and polyclonal sFLC patterns showed outcome associations across entities.	Mixed-entity design limits subtype-specific inference.
Landgren et al. 2010; Bibas et al. 2012 [37,38]	HIV-associated lymphoma risk	sFLC elevations preceded lymphoma in HIV-infected cohorts.	Risk-state biomarker; not diagnostic-phase specificity.
Tittle et al. 2015 [39]	HIV-associated lymphoma at diagnosis	sFLC did not correlate with histology or prognosis.	Supports separation of prediagnostic risk from diagnostic use.
Engels et al. 2012; Borrows et al. 2019 [43,44]	PTLD risk	Engels assessed prediagnostic risk; Borrows assessed patients at diagnosis with renal adjustment.	Different time points; neither supports stand-alone screening or diagnosis.
Hu et al. 2025 [45]	Population-based prediagnostic NHL	Higher kappa and lambda concentrations were associated with future NHL risk.	Between-group timing patterns do not demonstrate within-person clonal evolution or screening utility.

**Table 4:** Entity-specific interpretation and clinical boundaries.

Entity or Setting	Dominant or Plausible Pattern	Current Interpretive Role	Boundary
DLBCL	Polyclonal elevation is common; monoclonal subset may be biologically relevant.	Adjunctive baseline biological and risk context.	Not validated as an independent treatment-decision tool; not a ctDNA substitute.
cHL	Predominantly polyclonal immune-activation signal.	Inflammatory baseline risk context.	Not validated for monitoring or PET-adapted decisions.
FL/MZL	Low-frequency or contextual abnormalities; monoclonal patterns may reflect plasmacytic differentiation or coexisting clone.	Occasional contextual interpretation.	Not routine risk stratification.
CLL/SLL	Monoclonal, polyclonal, and ratio-only patterns; κ + λ recorded separately as an overlapping measure of total burden.	Early-disease risk context, especially TTFT research.	Does not replace iwCLL criteria, genetic risk tools, or MRD.
MCL	Heterogeneous monoclonal or polyclonal patterns.	Hypothesis-generating risk enrichment in selected cases.	Not validated in the BTK-inhibitor, CAR-T, or bispecific/T-cell-engager era.
WM/LPL	Monoclonal involved-chain excess.	Selected longitudinal adjunct when involved sFLC is measurable.	Does not replace IgM, M-spike, marrow assessment, or IWWM criteria.
T-cell lymphomas	Usually a polyclonal immune-activation signal.	Research-level immune-activation marker.	A monoclonal pattern should suggest coexisting B-cell/plasma-cell disease.
HIV-associated lymphoma	Prediagnostic polyclonal or cumulative immune activation.	Prediagnostic research context.	Not a diagnostic-phase subtype or prognosis marker.
PTLD	Polyclonal or cumulative activation; highly renal-sensitive.	Research context with renal and EBV assessment.	Tissue diagnosis remains mandatory.
Sparse-data entities	Insufficient entity-specific serum evidence.	Research framing only.	No routine role proposed for BL/HGBL, PBL, ALK-positive LBCL, PEL/HHV-8 disease, or PCNSL serum sFLC.

## Data Availability

No new patient-level data were created or analysed in this study. The search strategy, selection summary, and structured evidence extraction are provided in Supplementary File S1.

## Supplementary Materials

The following supporting information can be downloaded at: https://www.mdpi.com/article/10.3390/curroncol33100580/s1, Table S1: PubMed search and selection summary; Table S2: Structured extraction of PubMed-indexed clinical and translational reports included in the narrative synthesis.

## Abbreviations

AKI	acute kidney injury
ALK	anaplastic lymphoma kinase
ART	antiretroviral therapy
BL	Burkitt lymphoma
BTK	Bruton tyrosine kinase
CAR-T	chimeric antigen receptor T-cell therapy
cHL	classical Hodgkin lymphoma
CKD	chronic kidney disease
CLL/SLL	chronic lymphocytic leukemia/small lymphocytic lymphoma
CNS	central nervous system
COO	cell of origin
ctDNA	circulating tumor DNA
DLBCL	diffuse large B-cell lymphoma
EBV	Epstein–Barr virus
eGFR	estimated glomerular filtration rate
FLC	free light chain
FL	follicular lymphoma
HGBL	high-grade B-cell lymphoma
HHV-8	human herpesvirus 8
HIV	human immunodeficiency virus
IFE	immunofixation electrophoresis
IgM	immunoglobulin M
IPI	International Prognostic Index
IP-10	interferon-inducible protein 10
IWWM	International Workshop on Waldenström Macroglobulinemia
LBCL	large B-cell lymphoma
LC-MGUS	light-chain monoclonal gammopathy of undetermined significance
MCL	mantle cell lymphoma
MGUS	monoclonal gammopathy of undetermined significance
MIPI	Mantle Cell Lymphoma International Prognostic Index
MRD	measurable residual disease
MZL	marginal zone lymphoma
NHL	non-Hodgkin lymphoma
PBL	plasmablastic lymphoma
PCNSL	primary central nervous system lymphoma
PEL	primary effusion lymphoma
PET/CT	positron-emission tomography/computed tomography
PLCO	Prostate, Lung, Colorectal and Ovarian Cancer Screening Trial
PTLD	post-transplant lymphoproliferative disorder
sFLC	serum free light chain
SPEP	serum protein electrophoresis
TTFT	time to first treatment
WM/LPL	Waldenström macroglobulinemia/lymphoplasmacytic lymphoma

## References

[1] Schroeder H.W., Cavacini L. (2010). Structure and function of immunoglobulins. J. Allergy Clin. Immunol..

[2] Bhole M.V., Sadler R., Ramasamy K. (2014). Serum-free light-chain assay: Clinical utility and limitations. Ann. Clin. Biochem..

[3] Katzmann J.A., Clark R.J., Abraham R.S., Bryant S., Lymp J.F., Bradwell A.R., Kyle R.A. (2002). Serum reference intervals and diagnostic ranges for free kappa and free lambda immunoglobulin light chains: Relative sensitivity for detection of monoclonal light chains. Clin. Chem..

[4] Hutchison C.A., Plant T., Drayson M., Cockwell P., Kountouri M., Basnayake K., Harding S., Bradwell A.R., Mead G. (2008). Serum free light chain measurement aids the diagnosis of myeloma in patients with severe renal failure. BMC Nephrol..

[5] Einarsson Long T.E., Indridason O.S., Palsson R., Rognvaldsson S., Love T.J., Thorsteinsdottir S., Sverrisdottir I.S., Vidarsson B., Onundarson P.T., Agnarsson B.A. (2022). Defining new reference intervals for serum free light chains in individuals with chronic kidney disease: Results of the iStopMM study. Blood Cancer J..

[6] Giles H.V., Karunanithi K. (2024). Performance characteristics and limitations of the available assays for the detection and quantitation of monoclonal free light chains and new emerging methodologies. Antibodies.

[7] Lock R.J., Saleem R., Roberts E.G., Wallage M.J., Pesce T.J., Rowbottom A., Cooper S.J., McEvoy E.D., Taylor J.L., Basu S. (2013). A multicentre study comparing two methods for serum free light chain analysis. Ann. Clin. Biochem..

[8] Daves M., Piccin A., Roccaforte V., Lippi G. (2021). Comparison of Freelite and N-Latex serum free light chain assays: A critical review. Biochem. Med. (Zagreb).

[9] Schieferdecker A., Hörber S., Ums M., Besemer B., Bokemeyer C., Peter A., Weisel K. (2020). Comparison of three different serum-free light-chain assays—Implications on diagnostic and therapeutic monitoring of multiple myeloma. Blood Cancer J..

[10] Ribatti D., Tamma R., Annese T., Ingravallo G., Specchia G. (2022). Inflammatory microenvironment in classical Hodgkin’s lymphoma with special stress on mast cells. Front. Oncol..

[11] Thompson C.A., Maurer M.J., Cerhan J.R., Katzmann J.A., Ansell S.M., Habermann T.M., Macon W.R., Weiner G.J., Link B.K., Witzig T.E. (2011). Elevated serum free light chains are associated with inferior event-free and overall survival in Hodgkin lymphoma. Am. J. Hematol..

[12] Chiba S., Sakata-Yanagimoto M. (2020). Advances in understanding of angioimmunoblastic T-cell lymphoma. Leukemia.

[13] Delfau-Larue M.H., de Leval L., Joly B., Plonquet A., Challine D., Parrens M., Delmer A., Salles G., Morschhauser F., Delarue R. (2012). Targeting intratumoral B cells with rituximab in addition to CHOP in angioimmunoblastic T-cell lymphoma: A clinicobiological study of the GELA. Haematologica.

[14] Itzykson R., Le Garff-Tavernier M., Katsahian S., Diemert M.C., Musset L., Leblond V. (2008). Serum-free light chain elevation is associated with a shorter time to treatment in Waldenström macroglobulinemia. Haematologica.

[15] Leleu X., Moreau A.S., Weller E., Roccaro A.M., Coiteux V., Manning R., Nelson M., Leduc R., Robu D., Dupire S. (2008). Serum immunoglobulin free light chain correlates with tumor burden markers in Waldenström macroglobulinemia. Leuk. Lymphoma.

[16] Leleu X., Xie W., Bagshaw M., Banwait R., Leduc R., Roper N., Weller E., Ghobrial I.M. (2011). The role of serum immunoglobulin free light chain in response and progression in Waldenström macroglobulinemia. Clin. Cancer Res..

[17] Gertz M.A. (2025). Waldenström macroglobulinemia: 2025 update on diagnosis, risk stratification, and management. Am. J. Hematol..

[18] Treon S.P., Tedeschi A., San-Miguel J., Garcia-Sanz R., Anderson K.C., Kimby E., Minnema M.C., Benevolo G., Qiu L., Yi S. (2023). Report of consensus Panel 4 from the 11th International Workshop on Waldenström Macroglobulinemia on diagnostic and response criteria. Semin. Hematol..

[19] Pratt G., El-Sharkawi D., Kothari J., D’Sa S., Auer R., McCarthy H., Krishna R., Miles O., Kyriakou C., Owen R. (2022). Diagnosis and management of Waldenström macroglobulinaemia: A British Society for Haematology guideline. Br. J. Haematol..

[20] Maurer M.J., Cerhan J.R., Katzmann J.A., Link B.K., Allmer C., Zent C.S., Call T.G., Rabe K.G., Hanson C.A., Kay N.E. (2011). Monoclonal and polyclonal serum free light chains and clinical outcome in chronic lymphocytic leukemia. Blood.

[21] Morabito F., De Filippi R., Laurenti L., Zirlik K., Recchia A.G., Gentile M., Morelli E., Vigna E., Gigliotti V., Calemma R. (2011). The cumulative amount of serum-free light chain is a strong prognosticator in chronic lymphocytic leukemia. Blood.

[22] Tsai H.T., Caporaso N.E., Kyle R.A., Katzmann J.A., Dispenzieri A., Hayes R.B., Marti G.E., Albitar M., Ghia P., Rajkumar S.V. (2009). Evidence of serum immunoglobulin abnormalities up to 9.8 years before diagnosis of chronic lymphocytic leukemia: A prospective study. Blood.

[23] Clay-Gilmour A.I., Rishi A.R., Goldin L.R., Greenberg-Worisek A.J., Achenbach S.J., Rabe K.G., Maurer M.J., Kay N.E., Shanafelt T.D., Call T.G. (2019). Association of elevated serum free light chains with chronic lymphocytic leukemia and monoclonal B-cell lymphocytosis. Blood Cancer J..

[24] Glenn M.J., Madsen M.J., Davis E., Garner C.D., Curtin K., Jones B., Williams J.A., Tomasson M.H., Camp N.J. (2019). Elevated IgM and abnormal free light chain ratio are increased in relatives from high-risk chronic lymphocytic leukemia pedigrees. Blood Cancer J..

[25] Maurer M.J., Micallef I.N., Cerhan J.R., Katzmann J.A., Link B.K., Colgan J.P., Habermann T.M., Inwards D.J., Markovic S.N., Ansell S.M. (2011). Elevated serum free light chains are associated with event-free and overall survival in two independent cohorts of patients with diffuse large B-cell lymphoma. J. Clin. Oncol..

[26] Witzig T.E., Maurer M.J., Stenson M.J., Allmer C., Macon W., Link B.K., Katzmann J.A., Gupta M. (2014). Elevated serum monoclonal and polyclonal free light chains and interferon-inducible protein-10 predicts inferior prognosis in untreated diffuse large B-cell lymphoma. Am. J. Hematol..

[27] Kim Y.R., Kim S.J., Cheong J.W., Kim Y., Jang J.E., Lee J.Y., Min Y.H., Song J.W., Yang W.I., Kim J.S. (2014). Monoclonal and polyclonal gammopathy measured by serum free light chain and immunofixation subdivide the clinical outcomes of diffuse large B-cell lymphoma according to molecular classification. Ann. Hematol..

[28] Maiolo E., Alma E., Napodano C., Gulli F., Bellesi S., Cuccaro A., Pocino K., D’Alo’ F., Iachini M., Martini M. (2020). The prognostic impact of monoclonal immunoglobulin and free light chain secretion in diffuse large B-cell lymphoma. Leuk. Lymphoma.

[29] Jia Z., Li J., Liu J., Han W., Xu W., Lu X. (2025). Prognostic value of serum free light chain abnormalities in diffuse large B-cell lymphoma. Front. Oncol..

[30] Achenbach M., Bittenbring J.T., Ziepert M., Regitz E., Ott G., Rosenwald A., Pfreundschuh M., Altmann B., Held G. (2014). Elevated serum free light chains do not predict outcome of elderly patients with aggressive CD20^+^ B-cell lymphomas. Br. J. Haematol..

[31] Alaggio R., Amador C., Anagnostopoulos I., Attygalle A.D., Araujo I.B.O., Berti E., Bhagat G., Borges A.M., Boyer D., Calaminici M. (2022). The 5th edition of the World Health Organization Classification of Haematolymphoid Tumours: Lymphoid Neoplasms. Leukemia.

[32] Campo E., Jaffe E.S., Cook J.R., Quintanilla-Martinez L., Swerdlow S.H., Anderson K.C., Brousset P., Cerroni L., de Leval L., Dirnhofer S. (2022). The International Consensus Classification of Mature Lymphoid Neoplasms: A report from the Clinical Advisory Committee. Blood.

[33] Fu L., Zhou X., Zhang X., Li X., Zhang F., Gu H., Wang X. (2025). Circulating tumor DNA in lymphoma: Technologies and applications. J. Hematol. Oncol..

[34] Cheson B.D., Fisher R.I., Barrington S.F., Cavalli F., Schwartz L.H., Zucca E., Lister T.A., Alliance, Australasian Leukaemia and Lymphoma Group, Eastern Cooperative Oncology Group, European Mantle Cell Lymphoma Consortium (2014). Recommendations for initial evaluation, staging, and response assessment of Hodgkin and non-Hodgkin lymphoma: The Lugano classification. J. Clin. Oncol..

[35] Baethge C., Goldbeck-Wood S., Mertens S. (2019). SANRA—A scale for the quality assessment of narrative review articles. Res. Integr. Peer Rev..

[36] Bosmann M., Kossler J., Stolz H., Walter U., Knop S., Steigerwald U. (2010). Detection of serum free light chains: The problem with antigen excess. Clin. Chem. Lab. Med..

[37] Landgren O., Goedert J.J., Rabkin C.S., Wilson W.H., Dunleavy K., Kyle R.A., Katzmann J.A., Rajkumar S.V., Engels E.A. (2010). Circulating serum free light chains as predictive markers of AIDS-related lymphoma. J. Clin. Oncol..

[38] Bibas M., Trotta M.P., Cozzi-Lepri A., Lorenzini P., Pinnetti C., Rizzardini G., Angarano G., Caramello P., Sighinolfi L., Mastroianni C.M. (2012). Role of serum free light chains in predicting HIV-associated non-Hodgkin lymphoma and Hodgkin’s lymphoma and its correlation with antiretroviral therapy. Am. J. Hematol..

[39] Tittle V., Rayment M., Keeling E., Gabriel I., Yarranton H., Bower M. (2015). Serum-free light chains in HIV-associated lymphoma: No correlation with histology or prognosis. AIDS.

[40] Zemlin A.E., Ipp H., Rensburg M.A., Germishuys J.J., Esser M.M., Olivier M., Erasmus R.T. (2015). Serum free light chains in patients with HIV infection: Their association with markers of disease severity and antiretroviral use. J. Clin. Pathol..

[41] Noy A. (2019). Optimizing treatment of HIV-associated lymphoma. Blood.

[42] Baptista M.J., Hernandez-Rodriguez A., Martinez-Caceres E., Morgades M., Martinez-Picado J., Sirera G., Sancho J.M., Feliu E., Ribera J.M., Navarro J.T. (2017). Epstein–Barr viral loads and serum free light chains levels are potential follow-up markers of HIV-related lymphomas. Leuk. Lymphoma.

[43] Engels E.A., Preiksaitis J., Zingone A., Landgren O. (2012). Circulating antibody free light chains and risk of posttransplant lymphoproliferative disorder. Am. J. Transplant..

[44] Borrows R., Scheer A., Cockwell P., Braun F., Anagnostopoulos I., Riess H., Zimmermann H., Trappe R.U. (2019). Serum-free light chains adjusted for renal function are a potential biomarker for post-transplant lymphoproliferative disorders. Ann. Hematol..

[45] Hu W., Landgren O., Bassig B.A., Purdue M.P., Engels E.A., Lan Q., Rothman N. (2025). Pre-diagnostic serum concentrations of free light chains and risk of non-Hodgkin lymphoma in a population-based prospective study. Leuk. Lymphoma.

[46] Einarsson Long T., Rognvaldsson S., Thorsteinsdottir S., Sverrisdottir I.S., Eythorsson E., Oskarsson J.T., Indridason O.S., Palsson R., Aspelund T., Vidarsson B. (2025). New definition of light chain monoclonal gammopathy of undetermined significance. JAMA Oncol..

[47] Andersen L.S., Mæng C.V., Rögnvaldsson S., Einarsson Long T., Brieghel C., Hermansen E., Andersen M.N., Grønbæk K., Niemann C.U., Kristinsson S.Y. (2026). Revised criteria for light chain MGUS enhance diagnostic accuracy and risk stratification. Blood Cancer J..

[48] Pasotti F., Da Molin S., Bosoni T., Azzarà G., Liga G., Moriello G., Zaccaria B., Buoro S. (2026). Free light chain EQA: Analytical variability with clinical consequences—Data from 25 Italian EQA exercises (2021–2025). Clin. Chim. Acta.

[49] Furtado M., Shah N., Levoguer A., Harding S., Rule S. (2013). Abnormal serum free light chain ratio predicts poor overall survival in mantle cell lymphoma. Br. J. Haematol..

[50] Witzig T.E., Maurer M.J., Habermann T.M., Link B.K., Micallef I.N., Nowakowski G.S., Ansell S.M., Colgan J.P., Inwards D.J., Porrata L.F. (2014). Elevated monoclonal and polyclonal serum immunoglobulin free light chain as prognostic factors in B- and T-cell non-Hodgkin lymphoma. Am. J. Hematol..

[51] Hasenclever D., Diehl V. (1998). A prognostic score for advanced Hodgkin’s disease. N. Engl. J. Med..

[52] Schroers-Martin J.G., Advani R.H. (2024). The role of response-adapted therapy in the era of novel agents. Semin. Hematol..

[53] Martin W., Abraham R., Shanafelt T., Clark R.J., Bone N., Geyer S.M., Katzmann J.A., Bradwell A., Kay N.E., Witzig T.E. (2007). Serum-free light chain—A new biomarker for patients with B-cell non-Hodgkin lymphoma and chronic lymphocytic leukemia. Transl. Res..

[54] Solal-Celigny P., Roy P., Colombat P., White J., Armitage J.O., Arranz-Saez R., Au W.Y., Bellei M., Brice P., Caballero D. (2004). Follicular lymphoma international prognostic index. Blood.

[55] Gradowski J.F., Jaffe E.S., Warnke R.A., Pittaluga S., Surti U., Gole L.A., Swerdlow S.H. (2010). Follicular lymphomas with plasmacytic differentiation include two subtypes. Mod. Pathol..

[56] Davids M.S., Stilgenbauer S., Tam C.S. (2026). First-line treatment for CLL in the era of targeted therapy. Blood Cancer J..

[57] Hallek M., Cheson B.D., Catovsky D., Caligaris-Cappio F., Dighiero G., Döhner H., Hillmen P., Keating M., Montserrat E., Chiorazzi N. (2018). iwCLL guidelines for diagnosis, indications for treatment, response assessment, and supportive management of CLL. Blood.

[58] Jerkeman M., Aurer I., Campo E., Cheah C.Y., Clark J., Doorduijn J., Eyre T.A., Fehr M., Giné E., Gomes da Silva M. (2025). EHA–EU MCL network guidelines for diagnosis and treatment of mantle cell lymphoma. HemaSphere.

[59] Kwapisz D. (2021). Serum free light chains in solid organ transplant recipients. Clin. Transplant..

[60] Preiksaitis J., Allen U., Bollard C.M., Dharnidharka V.R., Dulek D.E., Green M., Martinez O.M., Metes D.M., Michaels M.G., Smets F. (2024). The IPTA Nashville Consensus Conference on Post-Transplant Lymphoproliferative Disorders after Solid Organ Transplantation in Children: III—Consensus Guidelines for Epstein–Barr Virus Load and Other Biomarker Monitoring. Pediatr. Transplant..

