# Supplementary material for: Serum Free Light Chains in Lymphoma: A Phenotype-First Framework for Biological Interpretation, Analytical Control, and Clinical Use
Source: Curr Oncol. 2026 Sep 28;33(10):580. doi: 10.3390/curroncol33100580 (PMC13703368; doi:10.3390/curroncol33100580)
Supplement: Supplementary file 1 [file curroncol-33-00580-s001.zip › Supplementary File S1.pdf]

Supplementary File S1

Search Strategy Selection Summary and Study Level Evidence Extraction

Serum Free Light Chains in Lymphoma: A Phenotype-First Framework for Biological Interpretation, Analytical Control, and Clinical Use

S1 Search Strategy

PubMed/MEDLINE was searched on 17 June 2026 for records published from 1 January 1980 through that date, without a language restriction. The search targeted circulating free light chains in lymphoma, chronic lymphocytic leukemia, Waldenström macroglobulinemia, and post-transplant lymphoproliferative disorders. Additional analytical, biological, classification, and practice sources were identified by backward and forward citation chaining. These additional sources were not counted as a second exhaustive database search.

Exact PubMed query

```
("free light chain"[Title/Abstract] OR "free light chains"[Title/Abstract] OR sFLC[Title/Abstract])
AND (lymphoma*[Title/Abstract] OR "chronic lymphocytic leukemia"[Title/Abstract] OR "chronic
lymphocytic leukaemia"[Title/Abstract] OR Waldenstrom*[Title/Abstract] OR "post-transplant
lymphoproliferative"[Title/Abstract] OR "posttransplant lymphoproliferative"[Title/Abstract]) AND
("1980/01/01"[Date - Publication] : "2026/09/17"[Date - Publication])
```

S2 Selection and Appraisal

Eligible primary evidence comprised human studies measuring circulating free light chains in lymphoma or CLL/SLL, clinically linked translational studies, and prediagnostic or transplant studies with a lymphoma-related endpoint. Analytical and renal-reference studies were retained when they materially affected interpretation. Case reports, conference abstracts, urine-only or cerebrospinal-fluid-only studies, and reports without a relevant circulating-FLC result were not used to support clinical applications. Reviews and consensus documents supplied analytical or clinical context rather than independent biomarker cohorts.

Selection and extraction were not independently duplicated. Reports were appraised for temporality, adjustment for established risk factors, renal confounding, assay comparability, missing data, cohort overlap, treatment era, and external validation. Data were recorded from published reports; information absent from a publication or indexed abstract was not inferred. Negative findings and numerical inconsistencies were retained. No formal risk-of-bias or certainty instrument was applied, and no meta-analysis was performed.

Table S1. PubMed search and selection summary.

Item	Detail
Database	PubMed/MEDLINE
Search date	Up to 17 June 2026
Publication range	1 January 1980 to 17 June 2026
Language restriction	None
Records retrieved	226
Records included in the narrative synthesis	27
Records not included after title and abstract screening	199
Duplicate removal	Not applicable to a single PubMed result set identified by unique PMID
Additional source identification	Backward and forward citation chaining for analytical, renal, biological, classification, and practice context
Synthesis	Structured narrative review; no meta-analysis

S3 Study Level Evidence Extraction

Table S2 summarizes the 27 PubMed-indexed reports from the search that were included in the narrative synthesis. The table preserves the distinction between prognostic, diagnostic-phase, prediagnostic, and treatment-monitoring questions. Cohorts that overlap are identified, and renal restriction, renal reference intervals, renal normalization, and statistical adjustment are reported separately.

Table S2. Structured extraction of PubMed-indexed clinical and translational reports included in the narrative synthesis.

Study and setting	Population and design	Era or treatment	Assay and definition	Renal handling	Analysis and principal result	Key limitation
Maurer et al. 2011 [25] — DLBCL	295 (76 + 219); Trial-linked cohort and prospective observational cohort	MER 2002–2008; trial 2006–2007; predominantly immunochemotherapy	Freelite; κ >19.4 or λ >26.3 mg/L; ratio outside 0.26–1.65	Creatinine recorded; normal-creatinine sensitivity analysis; no eGFR-specific classification	IPI-adjusted and individual IPI-component models; discrimination assessed. IPI-adjusted EFS HR 2.37 (1.58–3.55); OS HR 2.94 (1.85–4.77). C-index improvement reported.	Historical regimens; no management-impact trial. The 2014 report below overlaps.
Witzig et al. 2014 [26] — DLBCL	276; 256 in immunochemotherapy outcome analysis; Follow-up and translational reanalysis	Subset of the 2011 study population	Freelite; elevated κ/λ with abnormal ratio = monoclonal; elevated chain(s) with normal ratio = polyclonal	No new eGFR-specific model reported	Phenotype-stratified survival and biological analyses. Both elevation patterns associated with inferior EFS; monoclonal cases enriched for non-GCB surrogates.	Not an independent replication; tissue-based analyses available only in a subset.
Kim et al. 2014 [27] — DLBCL	175; Retrospective single-centre cohort	2010–2012; all R-CHOP	Freelite; reported κ >18.4 or λ >26.3 mg/L; ratio 0.26–1.65. MG also included positive IFE.	Renal-failure ratio 0.37–3.1; 10 elevated creatinine; no renal covariate in final model	Final overall-cohort Cox model: IPI high-intermediate/high and elevated FLC. Adjusted EFS HR 2.84 (1.29–6.25); OS association reported (P=.005).	Published OS HR 4.54 has CI 4.56–13.19, excluding its point estimate; not corrected. IHC subgroup counts also inconsistent.
Maiolo et al. 2020 [28] — DLBCL	106 (61 M-protein; 45 controls); Retrospective selected cohort; controls with similar age/IPI factors	2006–2017 registry; rituximab–CHOP	Freelite on Optilite; frozen plasma; κ >19.4 or λ >26.3 mg/L; ratio 0.26–1.65; ROC-derived κ >50 mg/L	No specific renal exclusion, interval or adjustment reported	IPI-based control selection; Kaplan–Meier/log-rank; no adjusted FLC model reported. FLC did not stratify M-protein-positive cases; κ >50 mg/L associated with worse outcome in controls (HR 5.7; 1.5–21.2).	Data-derived cut-off, selected sample; methods define EFS but results/figure also use PFS terminology; not an independently validated threshold.
Jia et al. 2025 [29] — DLBCL	259; survival analysis 191; Retrospective two-centre cohort	2017–2022; R-CHOP for survival cohort	Immunoturbidimetry with monoclonal antibodies; manufacturer/platform not specified; κ >19.4 or λ >26.3 mg/L; ratio 0.26–1.65; monoclonal defined by ratio	Renal-failure ratio interval 0.37–3.1 stated; six with elevated creatinine; no renal-adjusted outcome model reported	Cox analysis stated; independent FLC significance not established. Unadjusted three-year OS/PFS worse with abnormal FLC.	68 lost to follow-up; multivariable details limited; reported HR-associated intervals ambiguous and not reported in the indexed publication.
Achenbach et al. 2014 [30] — aggressive CD20-positive B-cell lymphoma	259 training; 155 independent validation; Biomarker analysis of prospective trials	RICOVER-60 CHOP-14 ± rituximab; validation all rituximab-treated; age 61–80	Freelite on SPA PLUS; elevated concentration and abnormal ratio assessed	CKD-EPI eGFR ≥60 mL/min/1.73 m² required	Cox models adjusted for individual IPI variables. Training elevated-FLC EFS HR 0.9 (0.6–1.5); OS 1.1 (0.6–1.9). Negative findings reproduced in validation.	Restricted age and preserved renal function; aggressive CD20-positive B-cell population, not unrestricted DLBCL.
Thompson et al. 2011 [11] — Hodgkin lymphoma	100 (93 cHL); Prospective observational cohort	2002–2008; 85% ABVD	Freelite; κ >19.4 or λ >26.3 mg/L; ratio 0.26–1.65	GFR compared; separate GFR-adjusted EFS analysis	IPS-adjusted EFS; insufficient deaths for adjusted OS. Adjusted EFS HR 3.02 (1.02–8.89).	Only 18 events and 12 deaths; abstract/body estimates differ; main-text adjusted estimate used.
Maurer et al. 2011 [20] — CLL/SLL	339; Prospective newly diagnosed cohort	2002–2008; pretreatment samples	Freelite; monoclonal, polyclonal, and ratio-only categories	Creatinine recorded; no eGFR-specific classification	Rai adjustment and composite clinical/biological risk-score adjustment. Composite-adjusted monoclonal TTFT HR 2.97 (1.75–5.06); elevated FLC OS HR 2.25 (1.04–4.86).	Limited events precluded unrestricted full-covariate modelling; not targeted-therapy validation.
Morabito et al. 2011 [21] — CLL	449; Treatment-naïve clinical cohort	Pretreatment; historical era	Freelite on Delta nephelometer; ratio 0.26–1.65; κ + λ sum	Ratio 0.37–3.1 applied for impaired creatinine clearance; not adjustment of cumulative concentration	Cox models with CD38, ZAP-70, IGHV, Binet stage and cytogenetics; both FLC measures compared. Joint model: cumulative FLC HR 2.3 (1.3–4.1), with cytogenetics, ZAP-70 and Binet stage; ratio lost independence.	Threshold printed as 60.6 mg/mL despite assay mg/L units; another HR/CI pair is inconsistent. Neither is silently corrected; cut-off not recommended.

Study and setting	Population and design	Era or treatment	Assay and definition	Renal handling	Analysis and principal result	Key limitation
Tsai et al. 2009 [22] — prediagnostic CLL	109 future CLL cases in the PLCO cohort; prospective prediagnostic serum study	Samples obtained up to 9.8 years before CLL diagnosis	Serum FLC ratio and concentrations; M-protein and immunoglobulins also assessed	Renal handling not reported in the indexed summary	Descriptive longitudinal timing. Abnormal ratio in 38%, M-protein in 13%, and polyclonal elevation in 16% before diagnosis.	No screening threshold or intervention analysis; case-only prediagnostic design.
Clay-Gilmour et al. 2019 [23] — familial/sporadic CLL and MBL	Familial CLL 154; sporadic CLL 302; familial MBL 87; unaffected relatives 263; reference population 15,396	Pretreatment serum; contemporary familial and sporadic cohorts	Monoclonal and polyclonal sFLC categories; reference-population comparison	Renal adjustment not reported in the indexed summary	Age-stratified and age/sex-adjusted logistic models. Monoclonal elevations were more frequent in CLL and in older MBL/relative groups.	Cross-sectional susceptibility comparison; no treatment or outcome endpoint.
Glenn et al. 2019 [24] — high-risk CLL pedigrees	Relatives in 23 high-risk CLL pedigrees compared with population controls	Familial-risk study	FLC ratio and immunoglobulin heavy-chain measures	Renal handling not reported in the indexed summary	Family clustering analyses. Elevated IgM and abnormal FLC ratio were more frequent among relatives.	Exploratory heritability and clustering; no validated individual risk threshold.
Furtado et al. 2013 [49] — MCL	Two small cohorts; uniform treated cohort n=20; Retrospective cohort plus phase II trial subset	Relapsed/refractory; lenalidomide	sFLC ratio; exact platform/limits not reported in the indexed publication	Not reported in the indexed abstract	Small-cohort survival comparisons. High baseline ratio associated with shorter OS in lenalidomide cohort.	Very small, regimen-specific; no contemporary BTK/CAR-T validation.
Itzykson et al. 2008 [14] — WM/LPL	42; Retrospective untreated cohort	Pretreatment; 28 symptomatic at FLC sampling	Freelite; involved chain matched to IgM isotype	Creatinine recorded; one severe chronic renal failure	Cox time-to-treatment model. Involved FLC retained a time-to-treatment association.	Baseline symptomatic disease partly drives association; small asymptomatic subgroup.
Leleu et al. 2008 [15] — WM	98; Clinical association study	Pretreatment/burden assessment; regimen not reported in the indexed publication	sFLC; study cut-off 60 mg/L; platform not reported in the indexed publication	Not reported in the indexed abstract	Univariable and multivariable burden-marker associations. sFLC associated with hemoglobin and β2-microglobulin; not simply IgM level.	Association with burden is not validation of monitoring or clinical utility.
Leleu et al. 2011 [16] — WM	48 (20 untreated; 28 relapsed/refractory); Prospective phase II biomarker subset; 48 of 63 trial patients	2006–2009 enrollment; bortezomib plus rituximab	Freelite; iFLC response ≥50% decrease or normalization; progression ≥25% rise from nadir to >ULN	Trial creatinine ≤2.5×ULN; no renal-adjusted biomarker model	Agreement and paired timing; landmark response/TTP analyses. Response 79% vs 60%; agreement κ=.38. In 27 dual responders: median 2.1 vs 3.7 months. Landmark response not significantly associated with TTP.	Excluded normal baseline iFLC (12), missing baseline (1), follow-up (2); flare ≥15% occurred and cannot distinguish progression by itself.
Witzig et al. 2014 [50] — mixed NHL	492 (453 B-cell; 34 T-cell; 5 NOS); Prospective observational cohort	Prospective MER cohort; heterogeneous management; recruitment dates not specified	Freelite; κ >19.4 or λ >26.3 mg/L; ratio 0.26–1.65	No uniform eGFR-stratified model	Overall models adjusted for age, stage, performance status and LDH; subtype analyses small. FL 237, MALT 62, MCL 50; T-cell EFS HR 1.17 (0.55–2.49).	Do not infer a validated subtype effect from the overall pooled association.
Martin et al. 2007 [53] — B-cell NHL and CLL	226 patients: 208 NHL and 18 CLL; stored-serum comparison	Predates contemporary molecular classification and targeted therapy	Nephelometric sFLC compared with protein electrophoresis and immunofixation	Renal handling not reported in the indexed summary	Descriptive detection study. An M-protein was detected in 54/226; FLC identified some proteins not found by electrophoresis/immunofixation.	Detection study without validated diagnostic specificity, prognostic adjustment, or management endpoint.
Landgren et al. 2010 [37] — prediagnostic HIV-associated NHL	66 cases; 225 controls; Nested matched case-control study	Source cohorts 1982–2005	Freelite; absolute concentrations/ULN categories; serum/plasma	Renal adjustment not central to reported model	Conditional logistic regression with matching. κ and λ elevation preceded NHL by up to 2–5 years.	Historical HIV/ART contexts; risk association does not establish actionable screening.
Bibas et al. 2012 [38] — prediagnostic HIV-associated lymphoma	46 cases; 46 controls; ICONA nested matched case-control study	Antiretroviral-naïve cohort at entry; subsequent ART considered	κ + λ sum; >2-fold ULN category; platform not reported in the indexed publication	Not reported in the indexed abstract	Conditional logistic regression; CD4 and HIV/ART context. Cumulative FLC associated with subsequent lymphoma.	This study directly evaluated the sum; clinical implementation was not validated.
Tittle et al. 2015 [39] — HIV-associated lymphoma at diagnosis	264; Retrospective/prospective diagnostic cohort	1996–2014; ART era	Monoclonal/polyclonal FLC categories; platform not reported in the indexed publication	Not reported in the indexed abstract	Histology and OS analyses. No significant association with subtype or OS.	Does not negate prediagnostic associations; addresses a different endpoint.
Zemlin et al. 2015 [40] — HIV infection	366 people with HIV; cross-sectional laboratory study	Eighty-nine percent receiving antiretroviral therapy	Kappa, lambda, and ratio; albumin and IgG measured concurrently	Renal function was not a principal adjustment variable in the indexed report	Concentrations correlated with viral load and IgG and inversely with CD4 count and albumin; ART was associated with lower concentrations.	Not a lymphoma cohort; informs immune-activation confounding.

Study and setting	Population and design	Era or treatment	Assay and definition	Renal handling	Analysis and principal result	Key limitation
Baptista et al. 2017 [42] — HIV-related lymphoma follow-up	Small exploratory HIV-related lymphoma follow-up series	Treatment and follow-up setting	sFLC assessed with Epstein-Barr virus load	Renal handling and assay details not reported in the indexed record	Exploratory serial association; the report proposed EBV load and sFLC as potential follow-up markers.	Small report with insufficient detail for a validated monitoring rule.
Engels et al. 2012 [43] — prediagnostic PTLD	29 cases; 57 controls; Nested case-control study	Prediagnostic samples mean 3.5 months before PTLD plus diagnostic samples	Freelite; monoclonal/polyclonal categories	Interpretation complicated by renal function	Matched risk associations. Prediagnostic polyclonal OR 4.2 (1.1–15).	Short lead time; wide intervals; not a screening validation study.
Borrows et al. 2019 [44] — PTLD at diagnosis	20 cases; 90 controls; 14 follow-up cases; Diagnostic case-control and exploratory follow-up	Pretreatment at PTLD diagnosis	Combined FLC; renal ratio range; cystatin-C normalization	Cystatin C normalization; renal dysfunction common	Diagnostic associations; exploratory response follow-up. Renal normalization strengthened case-control association.	Not a prediagnostic study; study-derived normalized cut-off requires validation.
Hu et al. 2025 [45] — prediagnostic NHL	292 cases; matched controls; PLCO nested case-control study	Prediagnostic samples; stratified by time to diagnosis	κ, λ, and pattern categories; platform not reported in the indexed publication	Not reported in the indexed abstract	Risk and time-stratified analyses. Higher concentrations associated with NHL; subtype/timing differences.	Between-group timing patterns do not prove within-person clonal evolution.
Kwapisz 2021 [59] — solid-organ transplantation	Narrative review of sFLC after solid-organ transplantation	Literature through publication in 2021	Multiple sFLC assays considered; methods described as non-interchangeable	Renal function identified as essential for interpretation	Narrative synthesis of PTLD, infection, inflammation, and immunosuppression studies; PTLD results were contradictory.	Review, not an independent cohort; early-onset PTLD hypothesis requires prospective validation.

Abbreviations: ART, antiretroviral therapy; CI, confidence interval; CLL, chronic lymphocytic leukemia; DLBCL, diffuse large B-cell lymphoma; EFS, event-free survival; eGFR, estimated glomerular filtration rate; FLC, free light chain; HR, hazard ratio; IFE, immunofixation electrophoresis; IPI, International Prognostic Index; MBL, monoclonal B-cell lymphocytosis; MCL, mantle cell lymphoma; NHL, non-Hodgkin lymphoma; OS, overall survival; PLCO, Prostate, Lung, Colorectal and Ovarian Cancer Screening Trial; PTLD, post-transplant lymphoproliferative disorder; sFLC, serum free light chain; WM/LPL, Waldenström macroglobulinemia/lymphoplasmacytic lymphoma.
